# Association of ITPA Genotype with Event-Free Survival and Relapse Rates in Children with Acute Lymphoblastic Leukemia Undergoing Maintenance Therapy

**DOI:** 10.1371/journal.pone.0109551

**Published:** 2014-10-10

**Authors:** Alenka Smid, Natasa Karas-Kuzelicki, Miha Milek, Janez Jazbec, Irena Mlinaric-Rascan

**Affiliations:** 1 Faculty of Pharmacy, University of Ljubljana, Ljubljana, Slovenia; 2 University Children's Hospital, University Medical Centre Ljubljana, Ljubljana, Slovenia; University of Sydney, Australia

## Abstract

Although the treatment of acute lymphoblastic leukemia (ALL) has improved significantly over recent decades, failure due to treatment-related toxicities and relapse of the disease still occur in about 20% of patients. This retrospective study included 308 pediatric ALL patients undergoing maintenance therapy and investigated the effects of genetic variants of enzymes involved in the 6-mercaptopurine (6-MP) metabolism and folate pathway on survival and relapse rates. The presence of at least one of the non-functional *ITPA* alleles (94C>A and/or IVS2+21A>C variant) was associated with longer event-free survival compared to patients with the wild-type *ITPA* genotype (p = 0.033). Furthermore, patients carrying at least one non-functional *ITPA* allele were shown to be at a lower risk of suffering early (p = 0.003) and/or bone marrow relapse (p = 0.017). In conclusion, the *ITPA* genotype may serve as a genetic marker for the improvement of risk stratification and therapy individualization for patients with ALL.

## Introduction

Treatment of pediatric acute lymphoblastic leukemia (ALL) has improved drastically over the last forty years, with the long term-survival rate for children diagnosed with ALL now such that approximately 85% survive for 5 years or more after diagnosis [1]. This great improvement can be attributed both to risk-adjusted treatment based on the identification of several biological and clinical prognostic factors which facilitated the definition of patient subgroups with different relapse risks, and the implementation of rationally designed phases in the treatment backbone of ALL [2]. Despite this remarkable progress, treatment failure due to treatment-related toxicities, which can be life threatening, and drug resistance, which leads to relapse, still occur in about 20% of patients [3], for whom there is a very low likelihood of long-term survival [1], [4], [5], [6].

Genetic polymorphisms in thiopurine-S-methyltransferase (*TPMT*) influence the toxicity of 6-mercaptopurine (6-MP), used as the backbone of the maintenance therapy of ALL, and thus represent one of the best examples of clinically important pharmacogenetic markers [7], [8]. Patients on 6-MP therapy exhibiting decreased TPMT activity have been shown to be at greater risk of developing toxic effects, due to undesirably high thioguanine nucleotides (TGN) accumulation in cells [9]. On the other hand, ultra-high enzyme activity can lead to superior 6-MP tolerability but also an increased risk of relapse [10]. Despite the very well characterized influences of genetic polymorphisms on TPMT activity, it is known that there are other genetic and non-genetic factors influencing it. Our previous study in ALL patients revealed that the presence of low-activity methylenetetrahydrofolate reductase (*MTHFR*) alleles contributes to 6-MP toxicity, presumably by limiting S-adenosylmethionine (SAM) synthesis [11]. Further molecular and functional studies carried out *in vitro* on several cancer cell lines have demonstrated that SAM is responsible for direct post-translational TPMT stabilization, resulting in increased TPMT activity [12]. Since folate and methionine pathways are crucial for SAM synthesis, other polymorphisms in enzyme-coding genes (such as 5-methyltetrahydrofolate-homocysteine methyltransferase reductase (*MTRR*), methylenetetrahydrofolate dehydrogenase 1 (*MTHFD1*), betaine–homocysteine S-methyltransferase (*BHMT*), and glycine N-methyltransferase (*GNMT*)) could influence TPMT activity and thus affect the safety and efficacy of 6-MP therapy. A recent study in ALL patients conducted by Stocco et al. [13] has shown that genetic variation in *PACSIN2* also influences TPMT activity and is significantly associated with the incidence of 6-MP-related gastrointestinal toxicity [13].

**Figure 1:**
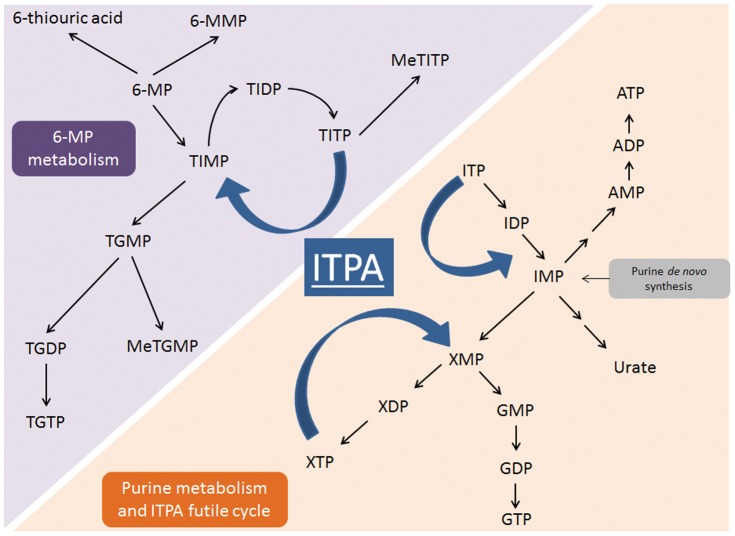
Schematic representation of the *ITPA* involvement in 6-MP metabolism and endogenous purine metabolism. 6-MP, 6-mercaptopurine; 6-MMP, 6-methylmercaptopurine; TIMP, thioinosine monophosphate; TIDP, thioinosine diphosphate; TITP, thioinosine triphosphate; MeTITP, methylthioinosine triphosphate; TGMP, thioguanosine monophosphate; TGDP, thioguanosine diphosphate; TGTP, thioguanosine triphosphate; MeTGMP, methylthioguanosine monophosphate; ITPA, inosine triphosphate pyrophosphatase; IMP, inosine monophosphate; XMP, xanthosine monophosphate; XDP, xanthosine diphosphate; XTP, xanthosine triphosphate; GMP, guanosine monophosphate; GDP, guanosine diphosphate; GTP, guanosine triphosphate; ITP, inosine triphosphate; IDP, inosine diphosphate; AMP, adenosine monophosphate; ADP, adenosine diphosphate; ATP, adenosine triphosphate.

Inosine triphosphate pyrophosphatase (ITPA) is an enzyme involved in 6-MP metabolism whose activity is determined by polymorphisms in the *ITPA* gene and is currently being extensively studied in relation to 6-MP treatment outcome. ITPA catalyses the pyrophosphohydrolysis of inosine triphosphate (ITP) into inosine monophosphate (Figure 1), and prevents the accumulation of potentially toxic compounds, such as ITP and deoxy-ITP or xanthosine triphosphate (XTP), that may be incorporated into RNA and DNA; in this way, it rescues the cell from apoptosis [14]. Although *ITPA* has been linked to 6-MP toxicity, different studies on patients with inflammatory bowel disease, liver transplant recipients and ALL patients showed inconsistent results [7], [15], [16], [17], [18], [19], [20], [21], [22], [23], [24]. Two SNPs in the *ITPA* gene, namely a non-synonymous C94A transition and the intronic IVS2+21A>C variant, with a frequency of approximately 6% and 13%, respectively, in Caucasians [25], have been correlated with defective enzyme activity [14], [26] leading to a higher risk of myelotoxicity and hepatotoxicity in pediatric ALL patients [7], [22], [27]. Furthermore, a recent Korean study suggested the *ITPA* 94C>A polymorphism might be associated with survival rate in pediatric ALL patients [21].

Despite intense efforts devoted to precise risk classification, patient characteristics at relapse reveal that more than half of relapsed patients were originally classified as non-high risk [28]. This highlights the need for the identification of additional prognostic markers.

In the present retrospective study we aimed to investigate the association of selected polymorphisms in genes involved in the folate and methionine pathway (*MTHFR* 677C>A, *MTHFR* 1298A>C, *MTRR* 66A>G, *MTHFD1* 1958G>A, *BHMT* 742G>A, *GNMT* 1298C>T), as well as polymorphisms of *PACSIN2* (rs2413739) and *ITPA* (94C>A and IVS2+21A>C), and long-term outcome and relapse rates in pediatric ALL patients undergoing maintenance therapy with 6-MP.

## Patients and Methods

### Study subjects

ALL patients diagnosed and treated at University Children's Hospital, University Medical Centre, Ljubljana, Slovenia from 1970 to 2006 were identified through the National Cancer Registry. Of 408 registered patients with childhood ALL, adequate documentation and intact genetic material was obtained from 308, which represented the final study group. Ethical approval for this study was obtained from the National Medical Ethics Committee of Slovenia (59/07/10) which waived the need for written informed consent, since the data was collected retrospectively and was analyzed anonymously.

The following therapy protocols were applied: from 1970 to 1983 the USA Pediatric Oncology Group (POG) protocols were used; from 1983 the therapy was changed to the German Berlin-Frankfurt-Muenster (BFM) protocols (BFM-83, -86, -90, -95 and IC2002). Maintenance therapy was included in all protocols and lasted from 1 to 3 years. The maintenance phase consisted of 6-MP taken daily per os (50 mg/m^2^) and weekly oral low-dose methotrexate (MTX) (20 mg/m^2^).

Patients' characteristics, such as gender, age at diagnosis, therapy protocol and risk group allocation, were obtained from their medical records. Patients were grouped according to age at diagnosis in 4 groups: (a) <1 year, (b) 1–6 years, (c) 6–12 years, and (d)>12 years, and according to treatment protocols to 3 groups: (a) POG protocol, (b) BFM83 or BFM86 protocol, and (c) BFM90, BFM95 or IC BFM2002 protocol.

### Event and risk group definition

The event in the calculation of event-free survival (EFS) probability was defined as any relapse, death, or secondary malignant neoplasm.

Patients were stratified into risk groups according to criteria described previously [29], [30], [31], [32]. The standard risk group included the low-risk group of POG, SR-L and SR-H groups of BFM-83 and SR groups of protocols BFM-86, -90 and -95. Intermediate or high-risk groups included the high-risk group of POG, R-group of BFM-86, MR groups of BFM-83, -90, -95 and HR groups of BFM-83, -90, -95 protocols.

### DNA extraction and genotyping

DNA extraction was performed as previously described [11], [33]. All the analyzed polymorphisms were determined by means of TaqMan chemistry using either the ABI Prism 7000 Sequence Detection system or the Roche LightCycler 480 system, in accordance with the manufacturers' instructions. The amount of DNA used in each individual assay was 10 ng. The TaqMan SNP Genotyping Assay part numbers are available in Table S1.

### Statistical analysis

The distributions of genotypes and possible deviations from the Hardy-Weinberg equilibrium were assessed by Fisher's exact test. Odds ratios, 95% confidence intervals (95% CI) and P values, showing the association of occurrence of relapse with studied genotypes, were calculated using logistic regression analysis. Covariates considered to be potential confounders, such as gender, age at diagnosis, ALL therapy protocol and risk group, were also placed in the model.

Kaplan-Meier event-free survival curves (EFS) for ALL patients were created, and the survival differences according to different genetic polymorphisms and prognostic variables were analyzed by log-rank test. The starting point for the observation time was date of diagnosis. Multivariate analysis was conducted using the Cox proportional-hazards regression model to analyze predictive factors. Cumulative incidence curves of the different types of relapses were estimated, adjusting for competing risks of the other events, and compared with the Gray's test. In both analyses, the observation time was censored at the last follow-up date if no event was observed.

To explore the independent and synergistic effects of the studied SNPs on the occurrence of relapse we performed a multifactor dimensionality reduction (MDR) analysis [34].

Statistical analyses were performed using R, IBM SPSS for Windows, version 21.0 and MDR software. For all tests, a 2-sided p<0.05 was considered statistically significant.

## Results

### Patients and genotyping

In total, 308 patients with childhood ALL were included in this study. Their clinical characteristics are presented in Table 1.

**Table 1:** Clinical characteristics of patients with childhood ALL (N = 308).

Characteristic	No	%
**Gender**		
Female	143	46.4
Male	165	53.6
**Mean age at diagnosis ± standard deviation**		5.9±4.3 years
<1 year	10	3.2
1<6 years	192	62.3
6<12 years	68	22.1
>12 years	38	12.3
**ALL therapy protocol, n (%)**		
POG	92	29.9
BFM 83/BFM 86	89	28.9
BFM 90/BFM 95/IC-BFM 2002	127	41.2
**Risk group, n (%)**		
Standard risk	134	43.5
Intermediate or high risk	94	30.5
undetermined	80	26

Abbreviations: POG, Pediatric Oncology Group; BFM, Berlin-Frankfurt-Muenster.

Genotyping was performed once the clinical data were extracted from patients' medical files and analyzed by researchers who were blinded to patients' medical data. All genotype distributions were in Hardy-Weinberg equilibrium (p>0.05). The variant allele frequencies of the analyzed polymorphisms are presented in Table 2 and summary of genotype data provided in Table S2.

**Table 2:** Frequency of the analyzed polymorphisms in patients with childhood ALL.

Gene	Variant	Wild type (N)	Heterozygous (N)	Homozygous variant (N)	Variant allele frequency (%)
TPMT	rs1142345 and rs1800460 (*3A)	290	18	0	3
TPMT	rs1800460 (*3B)	308	0	0	0
TPMT	rs1142345 (*3C)	305	3	0	0
MTHFR	rs1801133 (677C>A)	134	134	40	35
MTHFR	rs1801131 (1298 A>C)	143	131	34	33
MTRR	rs1801394 (66A>G)	59	140	109	58
MTHFD1	rs2236225 (1958G>A)	94	149	65	45
BHMT	rs3733890 (742G>A)	144	122	42	33
GNMT	rs10948059 (1289C>T)	89	139	80	49
PACSIN2	rs2413739†	111	139	55	41
ITPA	rs1127354 (94C>A)	268	40	0	6
ITPA	rs7270101 (IVS2+21A>C)	235	68	5	13

† PACSIN2 rs2413739 could not be determined in 3 patients.

Abbreviations: TPMT, Thiopurine S-methyltransferase; MTHFR, methylenetetrahydrofolate reductase; MTRR, 5-methyltetrahydrofolate-homocysteine methyltransferase reductase; MTHFD1, methylenetetrahydrofolate dehydrogenase 1; BHMT, betaine—homocysteine S-methyltransferase; GNMT, glycine N-methyltransferase; PACSIN2, protein kinase C and casein kinase substrate in neurons protein; ITPA, Inosine triphosphate pyrophosphatase.

### Analysis of patient survival and characteristics of relapsed patients

All the included patients were followed up for at least 5 years after diagnosis. The most significant improvement in overall, as well as event-free, survival rate was observed when patients were treated in accordance with later protocols, increasing from 58% (5-year OS) and 43% (5-year EFS) in patients treated by POG protocol to 91% (5-year OS) and 83% (5-year EFS) in patients treated by protocols BFM90/95/2002, as described in Table 3. Overall and event-free survival rates were not different in patients stratified to different risk groups (Table 3).

**Table 3:** Overall and event-free survival rates in patients with childhood ALL, according to treatment protocol and risk group stratification.

	5-year OS/EFS	Log-rank p	10-year OS/EFS	Log-rank p
**Overall survival rate ± SE %**				
** Treatment protocol (n)**				
POG (92)	60±5	<0.001	51±5	<0.001
BFM 83/86 (89)	74±5		71±5	
BFM 90/95/2002 (127)	91±2		91±3	
** Risk group (n)** 1				
Standard (94)	83±3	0.983	81±3	0.983
Intermediate or high (134)	84±4		80±4	
**Event-free survival rate ± SE %**				
** Treatment protocol (n)**				
POG (92)	43±5	<0.001	40±5	<0.001
BFM 83/86 (89)	64±5		61±5	
BFM 90/95/2002 (127)	83±3		82±3	
** Risk group (n)** 1				
Standard (94)	72±4	0.645	70±4	0.645
Intermediate or high (134)	76±4		74±5	

1 Risk group was not determined for 80 patients (those who were treated under POG protocol).

Abbreviations: POG, Pediatric Oncology Group; BFM, Berlin-Frankfurt-Muenster.

A total of 102 (33.1%) patients relapsed during the median follow-up time of 155 months, 5 patients (1.6%) died because of therapy-related complications and 6 patients (1.9%) developed secondary malignancy.

Of all relapsed patients, 36 (35.3%) were stratified to a standard risk group and only 21 (20.6%) to an intermediate or high risk group; 45 relapsed patients treated using POG protocols were not allocated to any risk group since stratification according to risk factors was not well established then. Clinical characteristics of relapsed patients are shown in Table 4.

**Table 4:** Clinical characteristics of relapsed patients (N = 102).

Characteristic	No	%
**Gender**		
Female	39	38.2
Male	63	61.8
**Time to relapse**		
Median time (range) 24 (4–163) months		
Very early relapse^1^	32	31.4
Early relapse^2^	43	42.1
Late relapse^3^	27	26.5
**Site of first relapse**		
Bone marrow	50	49.0
Combined^4^	21	20.6
Isolated EMD^5^	31	30.4
**Site of any relapse**		
Bone marrow (with or without EMD)	89	87.3
Only isolated EMD^5^	13	12.7
**Treatment protocol**		
POG	53	52.0
BFM 83/86	30	29.4
BFM 90/95/2002	19	18.6
**Initially assigned risk group**		
Standard risk	36	35.3
Intermediate or high risk	21	20.6
Undetermined	45	44.1

Abbreviations: POG, Pediatric Oncology Group; BFM, Berlin-Frankfurt-Muenster; EMD, extramedullary disease.

1 less than18 months from diagnosis.

2 more than 18 months after diagnosis and less than 6 months after the end of treatment.

3 more than 6 months after the end of treatment.

4 Bone marrow with or without extramedullary disease.

5 CNS/testes/Other (spinal channel, liver, iris, mesenterium, lymph nodes neck, labia major).

### Association of selected genotypes with relapse rate, overall and event-free survival

In search of novel biomarkers for the prediction of long-term outcomes and the occurrence of relapse, we addressed the involvement of selected polymorphisms in genes involved in 6-MP metabolism and folate and methionine pathways.

Multiple logistic regression analysis revealed that among all genes tested, only *ITPA* had a statistically significant effect on the occurrence of relapse, when the model was adjusted to treatment protocol group, age group and gender; namely, that *ITPA* wild-type patients were at higher risk of relapse than patients carrying at least one variant *ITPA* allele (odds ratio (OR) = 1.9; 95% CI = 1.1–3.4; p = 0.026).

**Figure 2:**
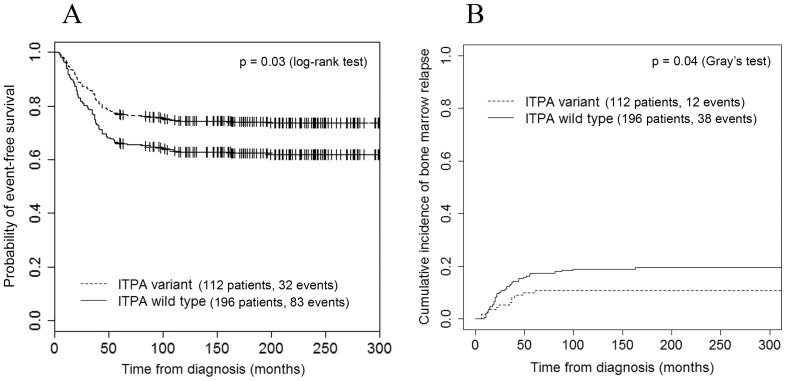
Event-free survival and cumulative incidence of bone marrow relapse by *ITPA* genotype (N = 308). **A**) *ITPA* wild-type genotype was shown to be an independent risk factor for lower event-free survival rate. 5-year EFS rates were 62% in wild-type patients and 74% in patients with at least 1 variant allele (log-rank test, p = 0.03). **B**) Cumulative incidences of bone marrow relapse in patients with wild-type *ITPA* and patients with at least one variant *ITPA* allele were 19.6% and 10.7%, respectively (Gray's test, p = 0.04).

In the univariate analysis using the Kaplan-Meier curve, the event-free survival (EFS) was affected by treatment protocol group, gender and *ITPA* genotype. EFS was lower in earlier protocols (43% in POG, 63% in BFM83/86 and 83% in BFM90/96/2002, p<0.001), in male patients as compared to female patients (60% and 72%, respectively; p = 0.022) and in the wild-type *ITPA* patients as compared to variant *ITPA* patients (62% and 74%, respectively; p = 0.030) (Figure 2A).

Multivariate analysis conducted with the Cox proportional-hazards regression model adjusted for treatment protocol group, gender and age group confirmed that among the genotypes tested, the *ITPA* 94CC/IVS2+21AA genotype was the only risk factor for lower EFS (HR 1.6, 95% CI 1.0–2.4; p = 0.033) (Table 5).

**Table 5:** Results of analysis of genotypes' influence on survival rates.

		*Univariate*		*Multivariate*	
		(Log rank)		(Cox regression)	
Genotype (No. of patients)	No. of events (% patients)	5-year EFS (%)	p	Hazard ratio (95% CI)	p
**Overall survival**					
** ** ***ITPA*** ** genotype (94C>A/IVS2+21A>C)**					
CC/AA (196)	59 (30%)	71%	0.164		
at least 1 variant allele^1^ (112)	25 (22%)	77%			
**Event-free survival**					
** ** ***ITPA*** ** genotype (94C>A/IVS2+21A>C)**					
CC/AA (196)	83 (42%)	62%	**0.030** *	1.6 (1.04–2.37)	**0.033** *
at least 1 variant allele^1^ (112)	32 (29%)	74%			
** ** ***TPMT*** ** genotype**					
1*/1* (287)	107 (37%)	79%	0.919		
1*/3* 2 (21)	8 (38%)	69%			
** ** ***MTHFR*** ** genotype (677C>A/1298 A>C)**					
CC/AA (31)	15 (48%)	58%	0.298		
at least 1 variant allele^3^ (277)	100 (36%)	67%			
** ** ***MTRR*** ** 66A>G**					
AA (59)	21 (36%)	66%	0.757		
AG/GG (249)	94 (38%)	66%			
** ** ***MTHFD*** ** 1958G>A**					
GG (94)	34 (36%)	68%	0.865		
GA/AA (214)	81 (38%)	65%			
** ** ***BHMT*** ** 742G>A**					
GG (144)	55 (38%)	67%	0.843		
GA/AA (164)	60 (37%)	67%			
** ** ***GNMT*** ** 1298C>T**					
CC (89)	32 (36%)	67%	0.841		
CT/TT (219)	83 (38%)	66%			
** ** ***PACSIN2*** ** rs2413739**					
CC (111)	40 (36%)	68%	0.727		
CT/TT (194)	73 (38%)	64%			

*denotes statistically significant difference.

1 *ITPA* genotype combinations with at least one variant allele (94CA/IVS2+21AA, 94CA/IVS2+21AC, 94CA/IVS2+21CC, 94CC/IVS2+21AC, 94CC/IVS2+21CC).

2 either a *1/*3A or *1/*3C *TPMT* genotype.

3 *MTHFR* genotype combinations with at least variant allele (677CC/1298AC, 677CT/1298AA, 677CC/1298CC, 677TT/1298AA, 677CT/1298AC, 677TT/1298AC).

Abbreviations: *TPMT*, Thiopurine S-methyltransferase; *MTHFR*, methylenetetrahydrofolate reductase; *MTRR*, 5-methyltetrahydrofolate-homocysteine methyltransferase reductase; *MTHFD1*, methylenetetrahydrofolate dehydrogenase 1; *BHMT*, betaine–homocysteine S-methyltransferase; *GNMT*, glycine N-methyltransferase; *PACSIN2*, protein kinase C and casein kinase substrate in neurons protein; *ITPA*, Inosine triphosphate pyrophosphatase.

### Influence of *ITPA* genotype on the site of relapse

Next, we aimed to explore whether the *ITPA* genotype has different effects in accordance with the relapse site. Patients experiencing relapse were divided into subgroups based on the site of the first relapse; namely, into those experiencing isolated bone marrow relapse (50 patients), combined bone marrow and extramedullary relapse (21 patients) and isolated extramedullary relapse (31 patients). The results of the multinomial logistic regression analysis adjusted to protocol treatment group, age group and gender revealed that wild-type *ITPA* patients were at higher risk of suffering medullary relapse (HR 2.5, 95% CI 1.2–5.2; p = 0.017) compared to variant *ITPA* patients, whereas the risk of experiencing a combined or isolated extramedullary relapse was not significantly different in patients with different *ITPA* genotypes. Furthermore, in competing risk regression analysis the risk of suffering a bone marrow relapse was two times higher for wild-type *ITPA* patients compared to variant *ITPA* patients (p = 0.040). The model was adjusted for age, gender and treatment protocol group. The cumulative incidence of bone marrow relapse in wild-type *ITPA* patients was 19.6%, while it was 10.7% in variant *ITPA* patients (Figure 2B).

### Influence of the *ITPA* genotypes on the time to relapse

Patients suffering relapse were divided into 3 subgroups in accordance with time to relapse criteria: very early (less than18 months from diagnosis), early (more than 18 months after diagnosis and less than 6 months after the end of treatment) and late relapse (more than 6 months after the end of treatment).

**Figure 3:**
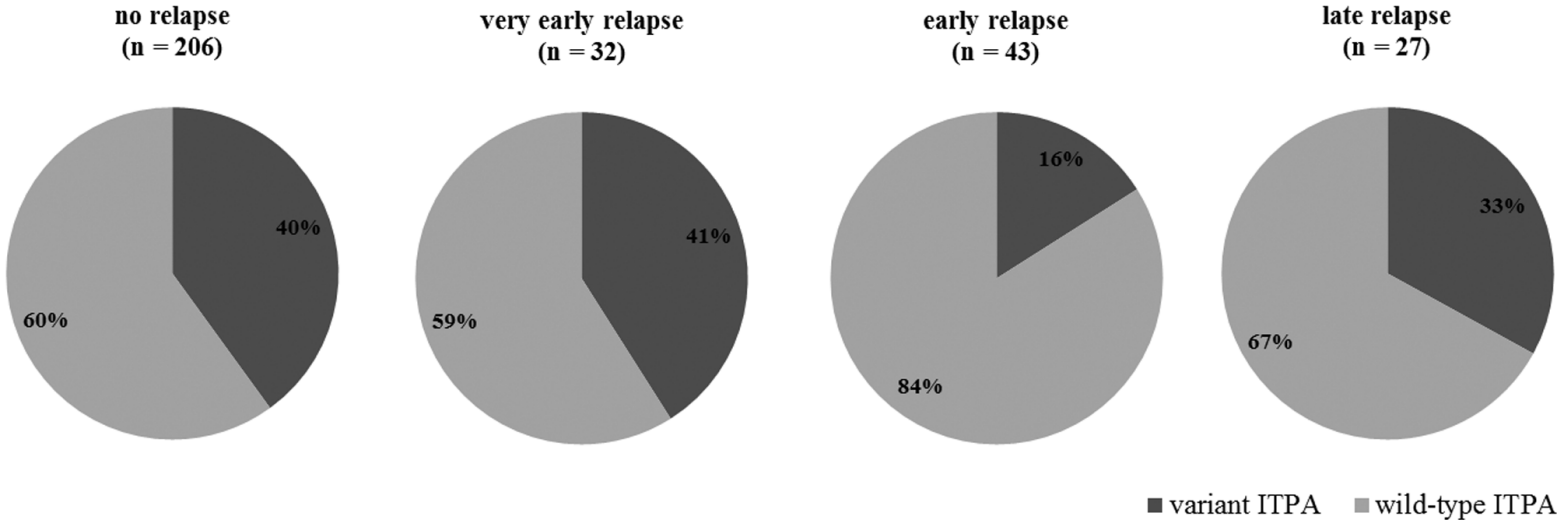
Influence of *ITPA* genotype on the incidence of relapses grouped according to time to relapse. Pie chart slices represent the percent of patients with different *ITPA* genotypes; wild-type *ITPA*: 94CC/IVS2+21AA ITPA genotype combination, variant *ITPA*: 94CA/IVS2+21AA, 94CA/IVS2+21AC, 94CA/IVS2+21CC, 94CC/IVS2+21AC, 94CC/IVS2+21CC ITPA genotype combinations. Very early relapse, <18 months after diagnosis; early relapse,>18 months after diagnosis and <6 months after the end of treatment; late relapse,>6 months after discontinuation of therapy (multinomial regression model adjusted to treatment protocol group, age group at diagnosis and gender; reference category  =  no relapse; p = 0.003).

The frequency of *ITPA* wild-type patients was significantly higher in the group of patients suffering early relapse (84%) compared to the group having no relapse (60%), very early relapse (59%) or late relapse (67%) (Fisher's exact test, p = 0.020). After adjustment to treatment protocol group, age and gender, the multinomial regression model confirmed that the effect of *ITPA* genotype was significant only for the occurrence of early relapse, such that *ITPA* wild-type patients are at higher risk of experiencing an early relapse than *ITPA* variant patients (HR 3.9, 95% CI 1.6–9.6; p = 0.003) (Figure 3).

### Gene-gene interactions

Gene-gene interactions between the variants and event-free survival or relapse were analysed using multifactor dimensionality reduction. There was no statistically significant multi-gene interaction model for event or relapse (data not shown).

## Discussion

The remarkable improvements in risk-directed treatments of childhood ALL observed over recent decades have resulted in an impressive increase in long term survival rates, currently approaching 85% in the developed world [35]. In contrast to the steadily improving outcome for patients with newly diagnosed ALL, little progress has been made in the treatment of relapsed ALL patients whose long-term survival rates range only from about 35% to 40% [1], [36], [37]. Relapse of the disease, therefore, remains a major concern in the treatment of ALL.

Due to the well-known importance of maintenance therapy for event-free and overall survival, this retrospective study, including all patients treated in Slovenia from 1970 to 2006 receiving at least one cycle of maintenance therapy, was conducted. We selected 8 candidate genes and identified 12 polymorphisms that could influence the metabolism of 6-MP, either by being directly involved in the metabolism or indirectly by altering TPMT activity, and evaluated their effects on survival and relapse rates.

We found that the inheritance of at least one of the non-functional *ITPA* alleles (94C>A and/or IVS2+21A>C) is associated with improved event-free survival and lower relapse rates in patients undergoing maintenance treatment for ALL.

ITPA is a human ITPase, a ‘house-cleaning’ enzyme, which protects cells from endogenous non-canonical nucleotides and, in the case of thiopurine therapy, from exogenous nucleotides, such as thio-ITP and methyl-thio-ITP, derived from thiopurines [38]. One possible explanation for the protective role of non-functional *ITPA* polymorphisms in relation to the relapse of ALL could be that the absence of functional ITPA activity can lead to the accumulation of non-canonical nucleotides that may cause DNA damage; such has also been demonstrated in an *in vitro* study on human HeLa cells with a knockdown *ITPA* gene [39]. Leukaemic blasts of the relapsed disease are more drug-resistant than blasts at initial diagnosis, which is probably the result of drug-resistant subclones being present at diagnosis or developing during therapy through the acquisition of additional genomic lesions [35]. It is possible that the action of 6-MP during the maintenance is partially reversed by the fully functional ITPA activity; this may then lead to the increased survival of leukemic blasts, which can, in turn, progress to relapse of the disease.

A more in-depth analysis of the impact of the *ITPA* genotype on relapse revealed that wild-type *ITPA* patients were at higher risk of suffering early relapse (HR 3.9, 95% CI 1.6–9.6; p = 0.003), whereas its effect on very early or late relapse was not statistically significant.

A study by Henderson et al. investigating the mechanism of relapse in ALL demonstrated that a shorter time interval to first relapse correlated with a higher quantity of the relapsing clone at diagnosis [40]. It is, therefore, possible that in the event of very early relapse occurring less than 18 months after the diagnosis, the amount of therapy-resistant sub-clones of leukemic blasts is too high for maintenance therapy to be effective in eradicating them. However, since the number of leukemic blasts in early relapse occurring more than 18 months after the diagnosis and less than 6 months after the end of treatment is lower, the influence of the *ITPA* genotype emerges by counteracting the 6-MP action. In the case of the wild-type *ITPA* genotype, the leukemic blasts are better protected against the therapy and are able to survive longer, resulting in a later expansion and onset of the relapse. On the other hand, the influence of the *ITPA* genotype diminishes again in late relapse occurring more than 6 months after the end of treatment. A possible explanation for this could lie in observed differences in gene expression between early and late relapse. Hogan et al. found that a set of genes involved in nucleotide biosynthesis and folate metabolism were specifically up-regulated in late relapse [41]. Another previous study by Zaza et al. has shown that the down-regulation of genes involved in purine metabolism correlates with the decreased *de novo* purine synthesis in EVT6/RUNX1 ALL and it has been postulated that this accounts for the chemosensitivity of this genetic subtype [42]. Overexpression in the blasts of patients who experience a late relapse offers an explanation for the resistance to antimetabolites and the lack of influence of the *ITPA* genotype in these patients.

The observation that *ITPA* genotype was associated only with bone marrow relapse could be explained by the role of the blood-brain and blood-testes barriers; these protect the leukemic blasts hidden in the CNS or testes (the so-called sanctuary sites) from the action of chemotherapeutics [43] and so the genes involved in the metabolism of anticancer drugs do not play such an important role in extramedullary relapse cases.

Several studies have previously been conducted studying the effects of *ITPA* polymorphisms on treatment outcome in ALL patients [7], [21], [22], [24], [25]; however, most of them focused on evaluating the effect of *ITPA* genotype on treatment-related toxicities and only a few on long-term survival. Stocco *et al.* reported that on maintenance therapy dosages, once the adjustment for *TPMT* had been made, the additional influence of *ITPA* genotype on 6-MP toxicity emerged [7]. No effect on long-term survival was seen therein. The effects of the combined *TPMT* and *ITPA* genotype on the mercaptopurine pharmacokinetics were also shown in a study by de Beaumais *et al.* [25] Furthermore, a study conducted in Indian ALL patients showed an independent role for both *TPMT* and *ITPA* in terms of association with the incidence of hematological toxicity [24]. The only study that associated *ITPA* genotype with survival rate was that conducted by Kim *et al.* in 100 Korean patients with pediatric ALL. They evaluated 18 loci in 16 candidate genes of pharmacogenetic interest, finding that the *ITPA* genotype, though not *TPMT*, had a significant effect on the event-free survival rate, which was lower in *ITPA* variants [21]. Like Kim *et al.*, we have not seen any effect of the *TPMT* genotype on event-free survival in pediatric ALL patients. However, and in contrast to their study, we found a different effect of the *ITPA* genotype on the event-free survival rate, such that it was lower in wild-type *ITPA* patients. A significant effect was observed most particularly on the occurrence of relapse, with patients carrying at least one *ITPA* variant allele being at lower risk of suffering early and/or bone marrow relapse. One possible explanation for the observed differences may be the fact that the study by Kim *et al.* was evaluating only one of the two non-functional polymorphisms of the *ITPA* gene, which is probably due to ethnic differences in *ITPA* genotype frequencies between Asian and Caucasian populations. Although the *ITPA* 94C>A polymorphism has been more frequently associated with 6-MP related toxicities, we believe that genotyping for *ITPA* IVS2+21A>C is also important due to its higher frequency in Caucasian populations. Indeed, when the two polymorphisms were evaluated independently, no significant correlation with event-free survival and/or relapse rate could be shown in our study. However, since both of these variants lower ITPA activity, we considered it rational to evaluate them together, thus producing only two variables: (a) wild-type *ITPA* and (b) variant *ITPA*. This could also explain why other studies, such as that by Stocco *et al.* evaluating only the *ITPA* 94C>A polymorphism, did not detect a significant effect of the *ITPA* genotype on survival or relapse rate [7]. Furthermore, since no association between *ITPA* genotype and treatment-related toxicity (especially febrile neutropenia) could be observed in the Korean study, they postulated that the lower survival rate in the variant group might not be influenced by accumulated toxic metabolites but by different factors, such as other genetic polymorphisms that were linked with *ITPA* 94C>A [21]. In contrast, our results support the hypothesis that variant *ITPA* alleles potentiate the effects of maintenance therapy by increasing the levels of toxic metabolites such that leukemic blasts are not protected and, therefore, unable to survive the attack of 6-MP. The effect is more pronounced in leukemic blasts in bone marrow and less so in blasts hidden in the sanctuary sites. Unfortunately, this hypothesis cannot be supported by measurements of toxic metabolites levels, since this was a retrospective study.

Our study evaluated a limited number of patients and, although we have taken into account - and adjusted our statistical models to - other factors influencing survival rates (the most important being treatment protocol), the effect of *ITPA* genotype should be evaluated in a larger cohort of patients treated according to the recent treatment protocols. In addition, we would like to point out that our conclusions are drawn from the analysis of DNA samples isolated from archival bone marrow smears prepared at the time of diagnosis of ALL. Since they contained a mixture of cells with different ratios of leukemic blasts and non-leukemic cells, the presence of hyperdiploid blasts and the potentially confounding effect of the acquisition of additional copies of chromosomes containing polymorphic genes on the concordance between the genotype and inherited phenotype could not be taken into account. Furthermore, additional molecular studies should be performed to elucidate the possible role of ITPA in relation to 6-MP therapy for the survival and expansion of leukemic blasts.

In summary, this study of pediatric ALL patients undergoing maintenance therapy shows that the *ITPA* genotype might be associated with event-free survival, with patients carrying at least one non-functional *ITPA* allele having higher event-free survival rates and a lower risk of suffering early and/or bone marrow relapse than wild-type *ITPA* patients.

## Supporting Information

Table S1 **List of TaqMan assays used for genotyping.** **Footnotes**: Abbreviations: TPMT, Thiopurine S-methyltransferase; MTHFR, methylenetetrahydrofolate reductase; MTRR, 5-methyltetrahydrofolate-homocysteine methyltransferase reductase; MTHFD1, methylenetetrahydrofolate dehydrogenase 1; BHMT, betaine–homocysteine S-methyltransferase; GNMT, glycine N-methyltransferase; PACSIN2, protein kinase C and casein kinase substrate in neurons protein; ITPA, Inosine triphosphate pyrophosphatase.
(DOCX)

Table S2 **Summary of genotype data of ALL patients analysed in the study.** **Footnotes**: ^1^ less than 18 months from diagnosis. ^2^ more than 18 months after diagnosis and less than 6 months after the end of treatment. ^3^ more than 6 months after the end of treatment. ^4^ percentage of patients within genotype group. Abbreviations: TPMT, Thiopurine S-methyltransferase; MTHFR, methylenetetrahydrofolate reductase; MTRR, 5-methyltetrahydrofolate-homocysteine methyltransferase reductase; MTHFD1, methylenetetrahydrofolate dehydrogenase 1; BHMT, betaine–homocysteine S-methyltransferase; GNMT, glycine N-methyltransferase; PACSIN2, protein kinase C and casein kinase substrate in neurons protein; ITPA, Inosine triphosphate pyrophosphatase.
(XLSX)
