# Supplementary material for: Association of ITPA Genotype with Event-Free Survival and Relapse Rates in Children with Acute Lymphoblastic Leukemia Undergoing Maintenance Therapy
Source: PLoS One. 2014 Oct 10;9(10):e109551. doi: 10.1371/journal.pone.0109551 (PMC4193781; doi:10.1371/journal.pone.0109551)
Supplement: Table S1 — List of TaqMan assays used for genotyping. Footnotes: Abbreviations: TPMT, Thiopurine S-methyltransferase; MTHFR, methylenetetrahydrofolate reductase; MTRR, 5-methyltetrahydrofolate-homocysteine methyltransferase reductase; MTHFD1, methylenetetrahydrofolate dehydrogenase 1; BHMT, betaine–homocysteine S-methyltransferase; GNMT, glycine N-methyltransferase; PACSIN2, protein kinase C and casein kinase substrate in neurons protein; ITPA, Inosine triphosphate pyrophosphatase. (DOCX) [file pone.0109551.s001.docx]

**Table S1: List of TaqMan assays used for genotyping**

| **Gene** | **Variant** | **TaqMan assay part number** |
| --- | --- | --- |
| TPMT | [rs1800460](http://www.pharmgkb.org/rsid/rs1800460) | C__30634116_20 |
| TPMT | rs1142345 | C_____19567_20 |
| MTHFR | rs1801133 | C___1202883_20 |
| MTHFR | [rs1801131](http://www.pharmgkb.org/rsid/rs1801131) | C____850486_20 |
| MTRR | rs1801394 | C___3068176_10 |
| MTHFD1 | rs2236225 | C___1376137_10 |
| BHMT | rs3733890 | C__11646606_20 |
| GNMT | rs10948059 | C__11425842_10 |
| PACSIN2 | rs2413739 | C___2503304_20 |
| ITPA | rs1127354 | C__27465000_10 |
| ITPA | rs7270101 | C__29168507_10 |

Abbreviations: TPMT, Thiopurine S-methyltransferase; MTHFR, methylenetetrahydrofolate reductase; MTRR, 5-methyltetrahydrofolate-homocysteine methyltransferase reductase; MTHFD1, methylenetetrahydrofolate dehydrogenase 1; BHMT, betaine--homocysteine S-methyltransferase; GNMT, glycine N-methyltransferase; PACSIN2, protein kinase C and casein kinase substrate in neurons protein; ITPA, Inosine triphosphate pyrophosphatase
